# Oxidation of Pharmaceuticals by Ferrate(VI)–Amino Acid Systems: Enhancement by Proline

**DOI:** 10.1021/acs.jpca.3c00134

**Published:** 2023-03-02

**Authors:** Virender K. Sharma, Junyue Wang, Mingbao Feng, Ching-Hua Huang

**Affiliations:** † Department of Environmental and Occupational Health, School of Public Health, Texas A&M University, College Station, Texas 77843-8371, United States; ‡ School of Civil and Environmental Engineering, Georgia Institute of Technology, Atlanta, Georgia 30332, United States

## Abstract

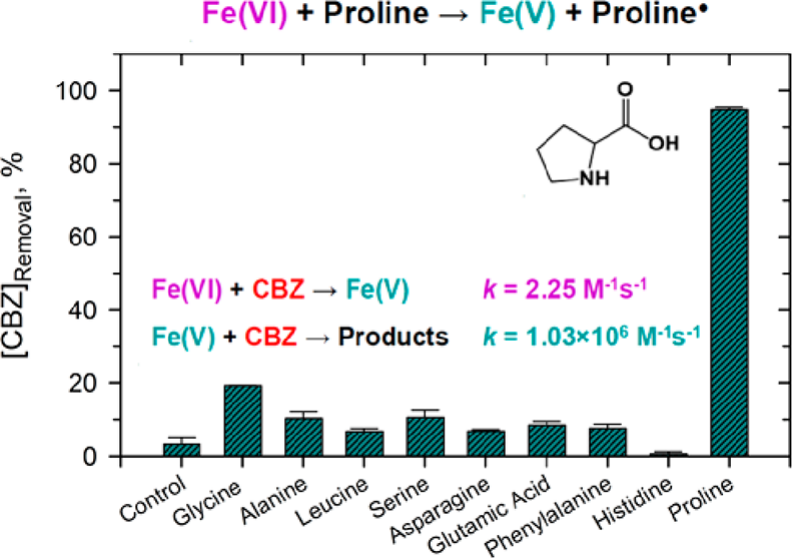

The occurrence of micropollutants in water threatens public health and ecology. Removal of micropollutants such as pharmaceuticals by a green oxidant, ferrate(VI) (Fe^VI^O_4_^2–^, Fe(VI)) can be accomplished. However, electron-deficient pharmaceuticals, such as carbamazepine (CBZ) showed a low removal rate by Fe(VI). This work investigates the activation of Fe(VI) by adding nine amino acids (AA) of different functionalities to accelerate the removal of CBZ in water under mild alkaline conditions. Among the studied amino acids, proline, a cyclic AA, had the highest removal of CBZ. The accelerated effect of proline was ascribed by demonstrating the involvement of highly reactive intermediate Fe(V) species, generated by one-electron transfer by the reaction of Fe(VI) with proline (i.e., Fe(VI) + proline → Fe(V) + proline^•^). The degradation kinetics of CBZ by a Fe(VI)–proline system was interpreted by kinetic modeling of the reactions involved that estimated the rate of the reaction of Fe(V) with CBZ as (1.03 ± 0.21) × 10^6^ M^–1^ s^–1^, which was several orders of magnitude greater than that of Fe(VI) of 2.25 M^–1^ s^–1^. Overall, natural compounds such as amino acids may be applied to increase the removal efficiency of recalcitrant micropollutants by Fe(VI).

## Introduction

Iron in different oxidation states ranging from 0 to +6 has tremendous potential in catalytic, synthetic, biological, and environmental reactions.^1−5^ Zero-valent iron (Fe(0)) in the reduction of halogenated compounds has been investigated.^6,7^ Iron(II) and iron(III) have applications in organic synthesis reactions.^8^ These low-valent iron in Fenton and Fenton-like reactions has been studied for many decades.^9−11^ High-valent iron (iron(IV) and iron(V)) has been the subject of numerous studies due to its roles in biological oxidation reactions, activation of inert carbon–hydrogen bonds, and decontamination of water.^12−15^ For example, iron(III) tetra-amidato macrocycle (Fe(III)-TAML) in a reaction with hypochlorite and hydrogen peroxide generates iron(IV) and iron(V) species to oxidize hydrocarbons and a wide range of pollutants.^16,17^ High-valent iron with an oxidation state of +6 commonly existing as FeO_4_^2–^ (Fe(VI)) in aqueous solution has been mainly studied in purifying water and wastewater.^18−22^

Fe(VI) performs multimodal actions in water purification, including disinfection, coagulation, and oxidation.^21,23−29^ Its inactivation of bacteria and viruses in water, wastewater, and hospital surfaces has been demonstrated.^29−31^ The importance of Fe(VI) oxidation in decreasing levels of disinfection byproducts in subsequent chlorination has been suggested.^32−35^ Significantly, the removal of lead, arsenic, cadmium, nickel, and phosphate using Fe(VI) has been shown.^36−39^ Most of the studies on Fe(VI) have been conducted on the oxidation of different pollutants, which included cyanides, thiocyanate, hydrogen sulfide, thiols, pesticides, phenols, anilines, sulfonamides, and tetracyclines.^3,40−44^ In the past few years, the focus has been on the oxidation of pharmaceuticals and personal care products by Fe(VI). Some of the pharmaceuticals showed sluggish reactivity with Fe(VI) that resulted in a decreased removal efficiency with a low oxidation capacity.^45,46^ Researchers thus have examined various activators to increase the removal efficiency of pharmaceuticals.^47^ Most of the studies were performed using a sulfite ion as an activator,^49−56^ while other activators such as peroxomonosulfate, thiosulfate, silica, biochar, and hydrochar have also been investigated.^48,49,51−56^

This paper presents our search for natural-based activators like amino acids to increase the removal efficiency of micropollutants by Fe(VI). We first investigated nine amino acids (aliphatic and aromatic) to activate Fe(VI) by carrying out removal experiments using carbamazepine (CBZ), which is an antiepileptic pharmaceutical and has been found as a micropollutant in different aquatic environments such as sewage treatment plant effluents.^28,57,58^ Among the studied amino acids, proline showed a distinct feature of enhanced oxidation of CBZ, which was then used to conduct further experimental and kinetic modeling studies to understand the activation mechanisms of Fe(VI).

## Experimental Methods

### Chemicals and Reagents

Carbamazepine (CBZ, >98% purity), sulfadimethoxine (SDM, >98% purity), trimethoprim (TMP, >98% purity), all test amino acids (i.e., glycine, alanine, serine, leucine, proline, phenylalanine, glutamic acid, asparagine, and histidine), phenyl methyl sulfoxide (PMSO), phenyl methyl sulfone (PMSO_2_), sodium thiosulfate, and sodium borate were purchased from Fisher Scientific (Austin, Texas, USA). A wet chemical procedure was applied to prepare solid potassium ferrate(VI) (K_2_FeO_4_),^59^ which had a purity of more than 90%. The solid K_2_FeO_4_ was dissolved in a 0.01 M sodium borate buffer solution. The concentration of Fe(VI) solution was determined by measuring the absorbance at 510 nm using a 1.0 cm path length and a molar absorption coefficient of ε_510nm_ = 1150 M^–1^ cm^–1^,^59^ with a UV–visible spectrometer (Thermo Scientific Co., USA). All reaction solutions were prepared using Milli-Q ultrapure water (>18 MΩ cm^–1^ resistivity, Millipore, Milford, USA).

### Degradation of Micropollutants in the Presence of Amino Acids

All experiments were performed in 100.0 mL glass beakers with at least duplicates under magnetic stirring. In studying the oxidation of CBZ, 10.0 mL of 10.0 μM CBZ was mixed with the same volume of 200.0 μM Fe(VI) to initiate the oxidation at pH 9.0. The effect of amino acids was evaluated by preadding them to 10.0 μM CBZ solution with a final concentration at 100.0 μM, and the reaction time was 60.0 s. CBZ is a recalcitrant pharmaceutical, and it has been found in complex matrices of wastewater containing organic matter and inorganic constituents. This suggests that carbamazepine does not hydrolyze in water.^28,57,58^ Additionally, the influence of different concentrations of proline (0–200.0 μM) was investigated as a function of the reaction time. The removal of PMSO and formation of PMSO_2_ by the Fe(VI)/proline system were conducted by spiking 5.0 μM PMSO into the reaction solutions at pH 9.0. At predetermined time intervals, 1.0 mL of the reaction solution was collected and quenched using a 1.0 M thiosulfate solution. The prepared samples were stored at 4 °C before analysis.

### Analytical Methods

CBZ, TMP, SDM, PMSO, and PMSO_2_ were determined using an Ultimate 3000 ultra-high-performance liquid chromatograph (UHPLC) (ThermoFisher Scientific) with a diode array detector. Chromatographic measurements were carried out on a RESTEK Ultra C_18_ column (4.6 mm × 250 mm, particle size of 5 μm). In analyzing CBZ, the column temperature was set at 30 °C and the mobile phase was 0.05% phosphoric acid in water (A) and methanol (B). The composition of A and B in the mobile phase was 30 and 70%, respectively. The wavelength of the detector was set at 284 nm. The HPLC conditions of other analytes are listed in Table S1.

### Determining Reaction Rate Constants between Fe(VI) and Amino Acids

The reactivity of Fe(VI) and amino acids was evaluated at pH 9.0 using stopped-flowed experiments under pseudo-first-order conditions (i.e., [amino acid] ≫ [Fe(VI)]). Specifically, 200.0 μM Fe(VI) and 2.0 mM amino acid solutions were rapidly mixed, and the kinetic traces were recorded at 510 nm with six replicate runs. The data obtained from the stopped-flow spectrophotometer (SX-20, Applied Photophysics, Surrey, UK) were processed via the nonlinear least-squares algorithm.

### Modeling

The degradation of CBZ by Fe(VI) and Fe(VI)–proline systems was modeled with reactions R1–R11 (Table 1, more discussion later) using the Kintecus program 4.55.31. Briefly, reaction R1 was first simulated by CBZ degradation in the absence of proline by the “FIT:2:3:FITDATA.TXT” command, where the initial proline concentration was set at zero. Then reactions R4 and R6 were simulated by CBZ degradation in the presence of proline (25.0–200.0 μM). The goodness-of-fit between the simulation and experimental data was quantified by calculating the normalized root-mean-square deviation (RMSD) (Table S3).

**Table 1:** Reactions in the Fe(VI)–Proline System and the Second-Order Rate Constants at pH 9.0

reaction no.	reactions	*k* (M^–1^ s^–1^)	ref
R1	Fe(VI) + CBZ → Fe(III) + products	2.25	simulated
R2	Fe(VI) + H_2_O → Fe(IV) + H_2_O_2_	negligible	(51)
R3	Fe(VI) + H_2_O_2_ → Fe(IV) + O_2_	negligible	(51)
R4	Fe(VI) + proline → Fe(V) + Pro•	7.1 × 10^1^	simulated
R5	Fe(VI) + Pro• → Fe(V)	1.0 × 10^9^	estimated from ref ^61^
R6	Fe(V) + CBZ → Fe(III) + products	(1.03 ± 0.21)× 10^6^	simulated
R7	Fe(V) + proline → Fe(III)	2.0 × 10^5^	estimated from ref ^60^
R8	Fe(V) + H_2_O → Fe(III) + H_2_O_2_	5.0	(51)
R9	2 Fe(V) → 2 Fe(III) + 2 H_2_O_2_	1.5 × 10^7^	(51)
R10	Fe(V) + H_2_O_2_ → Fe(III) + O_2_	4.0 × 10^5^	(51)
R11	Pro• + Pro• → products	5.1 × 10^8^	(67)

## Results and Discussion

### Degradation of Pharmaceuticals in the Presence of Amino Acids

**Figure 1:**
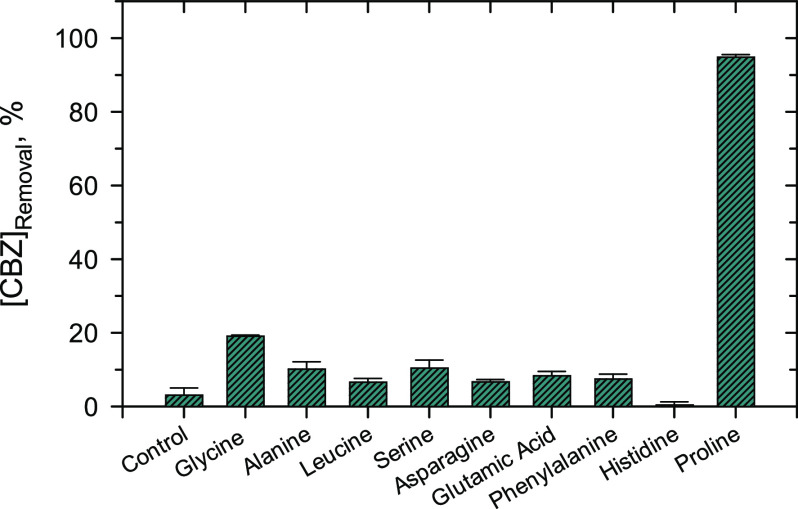
Removal of carbamazepine (CBZ) by Fe(VI)–amino acid systems. Experimental conditions: [CBZ] = 5.0 μM, [Fe(VI)] = 100.0 μM, [amino acid] = 100.0 μM, reaction time = 60 s, pH 9.0 (10.0 mM borate buffer).

In this study, different amino acids, glycine (Gly), alanine (Ala), leucine (Leu), serine (Ser), asparagine (Asn), glutamic acid (Glu), phenylalanine (Phe), histidine (His), and proline (Pro), were added in a solution of Fe(VI) and CBZ at pH 9.0. As seen in Figure S1, the studied amino acids differ in their structures such as aliphatic (Gly, Ala, and Leu), hydroxy (Ser), carboxamide (Asn), monoamine dicarboxylic (Glu), aromatic (Phe), diamino dicarboxylic (His), and cyclic (proline) groups. The degradation of CBZ by Fe(VI) alone in a 60.0 s reaction time was 3.2%. However, with the addition of amino acids, increased degradation of CBZ in 60.0 s was observed (Figure 1). In the presence of aliphatic amino acids, the CBZ removal was in the range of 10.2–21.2% with Gly showing the highest increase. Ser, Asn, and Glu had removals of 15.0, 17.7, and 7.6%, respectively. Aromatic amino acid (i.e., Phe) had a removal of 13.7%. His in the Fe(VI)–CBZ solution yielded the removal of ∼1%. The maximum removal of 70.6% was seen in the presence of proline, a cyclic amino acid. The comparative results of the removal of CBZ in Figure 1 suggested that the increased oxidation of CBZ may be influenced by diamino and cyclic amino acids (His and proline).

**Figure 2:**
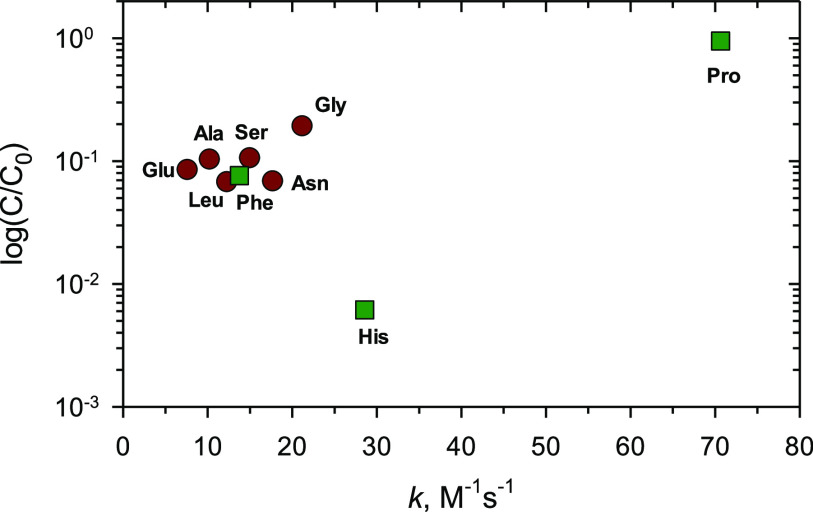
Correlation of removal efficiency (%) of CBZ by Fe(VI)–amino acid after 60 s with a second-order rate constant (*k*, M^–1^ s^–1^) of the reaction of amino acids by Fe(VI) at pH 9.0.

Fe(VI) may react with amino acid (AA) to produce a highly reactive species, Fe(V) (reaction 1). The occurrence of reaction 1 of the high-valent iron species via a one-electron transfer has been examined experimentally.^60,61^ The generated Fe(V) subsequently oxidizes CBZ to yield increased removal efficiency via reaction 2. In the absence of AA, the reaction of Fe(VI) with CBZ is slow, which does not generate a sufficient amount of Fe(V) species to result in significant removal of CBZ. Fe(V) may also be consumed by AA (reaction 3):
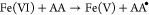
1
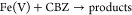
2
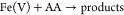
3It appears that the rate of reaction 1 is imperative to give the Fe(V) species and thus the increased degradation of CBZ. We have therefore determined the reactivity of Fe(VI) with AA at pH 9.0 in borate buffer. The obtained second-order rate constants (*k*, M^–1^ s^–1^) are given in Table S2. The plot of the logarithm (*C*/*C*_0_) versus the reactivity of Fe(VI) with AA (i.e., *k*) is shown in Figure 2. No apparent trend, except the proline, was noticed. With the increase of *k* by almost three times from 7.7 ± 0.1 to 21.2 ± 0.2 M^–1^ s^–1^, no corresponding increase in removal of CBZ could be obtained. Significantly, His, which had a *k* value of 28.5 ± 0.2 M^–1^ s^–1^ had no significant increase. Proline with the highest *k* of 70.6 ± 0.4 M^–1^ s^–1^ had the highest enhancement of CBZ removal (Figure 2). Even though other AAs can also react with Fe(VI) to produce Fe(V), they did not show much increase in removal even with an increased value of *k*, indicating not only the rate of reaction 1 to generate Fe(V) is important but also the nature of AA may have a role in the observed results of Figure 1. This finding is consistent with previous results on the removal of pharmaceuticals by Fe(VI) in the presence of amines and creatinine (2-amino-1-methyl-5*H*-imidazol-4-one).^42,62^

The role of proline in enhancing the oxidation of other micropollutants such as antibiotics was also explored. We tested trimethoprim (TMP) and sulfadimethoxine (SDM), which are antibiotics and have been found in aquatic systems.^63,64^ When only Fe(VI) was applied to TMP and SDM, the removal in the 60.0 s reaction time was 3.0 and 4.0%, respectively. However, when proline existed in the reaction, TMP could be removed completely, while the removal of SDM was >90%. This suggests that the Fe(VI)–proline system can be applied to remove micropollutants with greater efficiency than otherwise possible using Fe(VI).

### Plausible Mechanisms of Enhancement in Fe(VI)–Proline Systesm

**Figure 3:**
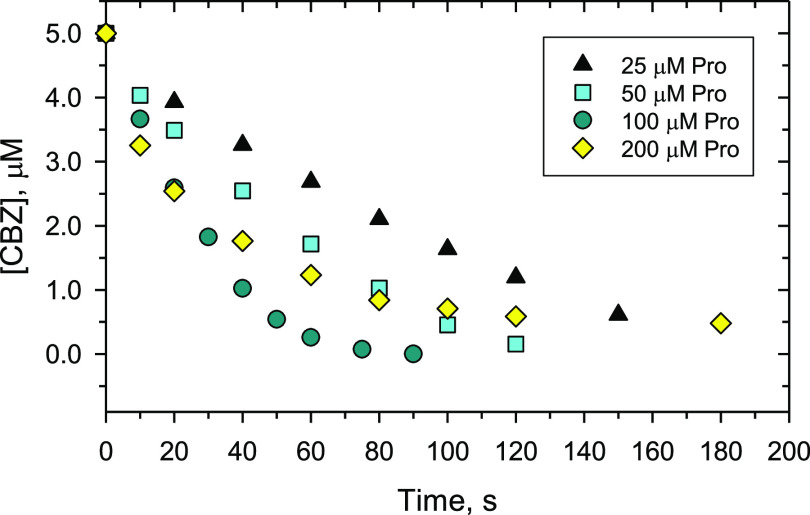
Removal of CBZ by Fe(VI)-proline system at different concentrations of proline. Experimental conditions: [CBZ] = 5.0 μM, [Fe(VI)] = 100.0 μM, [proline] = 25.0–200.0 μM, reaction time = 60 s, pH 9.0 (10.0 mM borate buffer)).

In the initial set of experiments, we investigated the effect of proline concentration on the removal rate of CBZ. When the proline concentration increased from 25 to 100 μM, the CBZ removal rate increased correspondingly (Figure 3). For example, in 60.0 s, the removal percentages of CBZ were 46.4, 65.7, and 94.0% for 25, 50, and 100 μM proline, respectively. However, with the further increase in the concentration of proline to 200 μM, the removal rate of CBZ decreased to 84.1% in 60 s. It appears that the high concentration of 200 μM proline could produce high amounts of Fe(V)/Fe(IV) species, but the possibility of inhibitory reactions did not allow Fe(V) to react with CBZ. The negative influence of such reactions is understood through kinetic modeling of the involved reactions and is described below.

**Figure 4:**
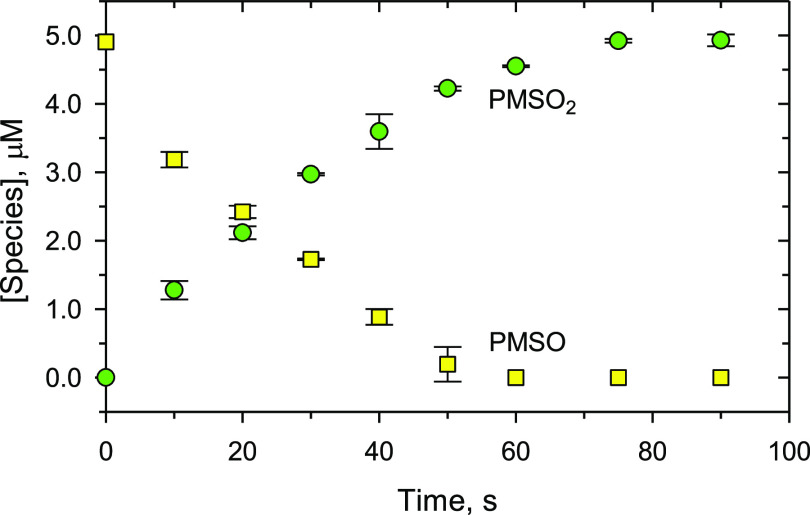
Oxidation of PMSO and formation of PMSO_2_ in the Fe(VI)–proline system. Experimental conditions: [PMSO] = 5.0 μM, [Fe(VI)] = 100.0 μM, [proline] = 100.0 μM, pH 9.0 (10.0 mM borate buffer).

Next, the formation of Fe(V) through reaction 1 in the Fe(VI)–proline–CBZ system was investigated experimentally using a probe molecule, PMSO. High-valent iron species selectively convert PMSO to PMSO_2_,^65,66^ indicating Fe(V) generation in the system. As shown in Figure 4, the formation of PMSO_2_ was seen, and there was a stoichiometric transformation from PMSO to PMSO_2_. This further supports that the generated Fe(V) from the reaction of Fe(VI) with proline caused the enhanced oxidation of CBZ. The nearly 100% conversion from PMSO to PMSO_2_ also confirmed that high-valent iron was the predominant reactive species in the system, while the contribution from other radicals was negligible.

Finally, the quantitative understanding of the enhancement of CBZ degradation by proline-activated Fe(VI) was done through a kinetic model, which was built upon 11 reactions (Table 1).^51,60,61,67^ In Table 1, we have written proline as Pro without emphasizing the equilibrium species of proline. The p*K*_a_ of the proline is 10.60,^61^ which suggests that protonated species of proline dominate at pH 9.0. However, for the reactivity of Fe(VI) with proline, the equilibrium of Fe(VI) needs to be considered, too (p*K*_a_ = 7.3).^21^ The reaction of Fe(VI) with amino acids involves one proton.^60,61^ This proton could be from either Fe(VI) or proline. In other words, there is proton ambiguity in the reaction of Fe(VI) with proline, which has not been resolved in the literature. We have therefore reasoned not to write a particular form of proline or also the Fe(VI) in the reactions given in Table 1.

Initially, reactions of Fe(VI) and CBZ without proline were considered (R1–R3). Fe(VI) reacts with CBZ to give products (R1). The slow reaction of Fe(VI) with water may also occur, which gives Fe(IV) and hydrogen peroxide (R2).^68^ Fe(VI) can then react with hydrogen peroxide to form Fe(IV) and oxygen (R3).^69^ In the presence of proline, additional reactions R4–R8 would occur. The reaction of Fe(VI) with proline gives Fe(V) and proline radical (Pro^•^) (R4). Fe(VI) can react with Pro• to generate another Fe(V) atom (R5). The formed Fe(V) through R4 and R5 reacts with CBZ (R6) to cause enhanced oxidation. Other possible reactions that give inhibitory influence on the oxidation of a target pollutant would be the Fe(V) reaction with proline (R7), its self-decomposition in water (R8 and R9),^70^ and reaction with hydrogen peroxide (R10).^69^ If Pro^•^ does not react with Fe(VI), it can be decomposed by another radical (R11). In the model, we have not considered the reaction of Pro^•^ with CBZ, which may possibly cause the degradation of CBZ in the Fe(VI)–proline–CBZ system. The Pro^•^ would be consumed by Fe(VI) rather than reacting with CBZ because (1) the rate constant of the reaction with Fe(VI) with Pro^•^ is of the order of 10^8^–10^9^ M^–1^ s^–1^, which is expected to be much higher than the reaction of an organic radical with an organic compound like CBZ,^60,61^ and (2) the concentration of Fe(VI) is 20 times higher than that of CBZ. Overall, the reaction of the Pro^•^ with CBZ is low and would not contribute to the decay of CBZ in the studied system.

**Figure 5:**
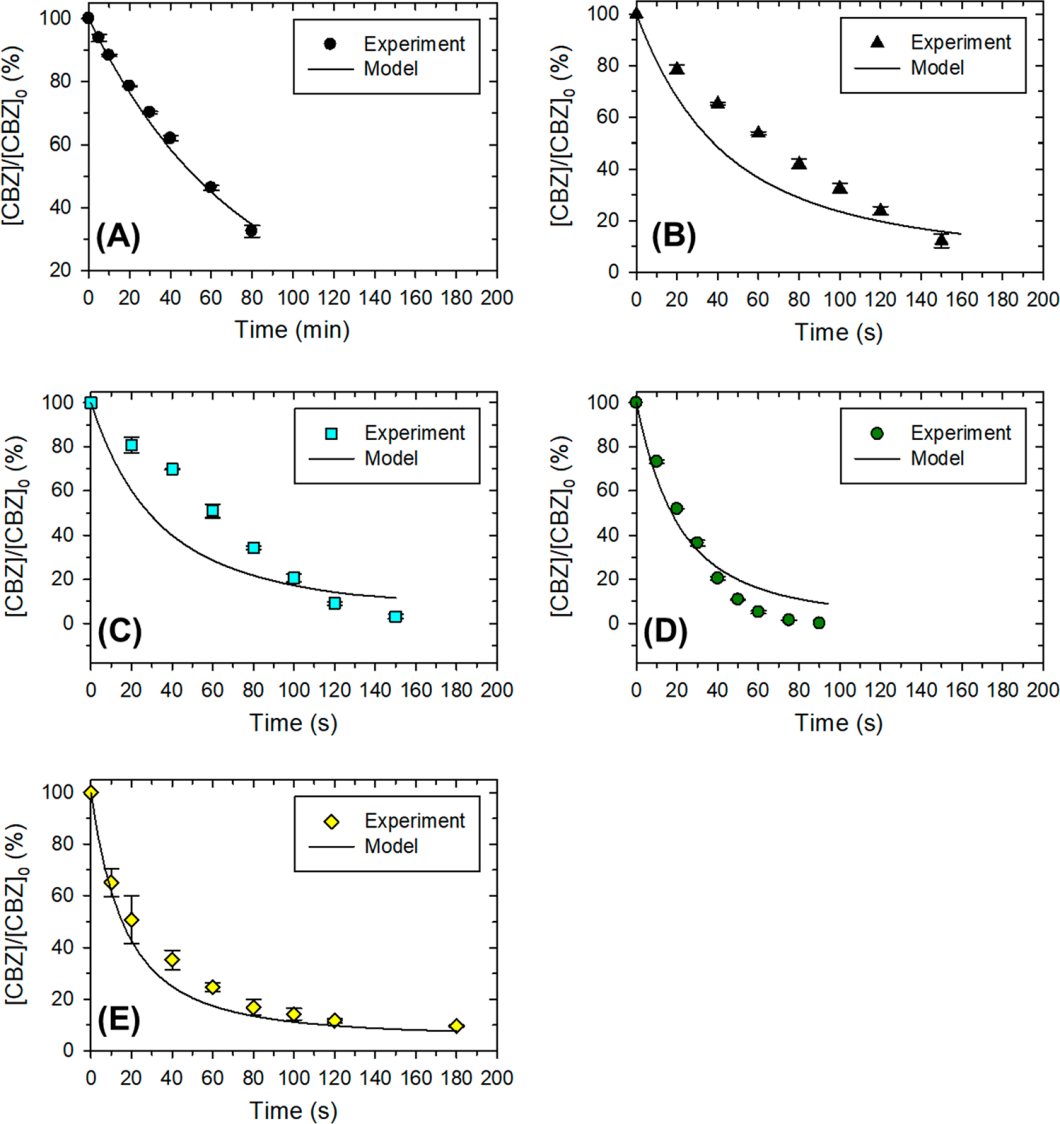
Degradation of CBZ by Fe(VI) at pH 9.0 with and without proline. Experimental conditions: [CBZ] = 5.0 μM, [Fe(VI)] = 100.0 μM, pH 9.0 (10.0 mM borate buffer), [proline] = 0 (A), 25 μM (B), 50 μM (C), 100 μM (D), 200 μM (E).

The results of the modeling of the experimental data in the absence of proline are presented in Figure 5. Reactions R2 and R3 were not considered in our study, similar to the earlier studies, in which Fe(VI) decay by R2 and R3 was negligible at pH 9.0. In the absence of proline, Fe(VI) oxidized CBZ slowly, with a second-order rate constant simulated at 2.25 M^–1^ s^–1^ (Figure 5A). The experimental degradation of CBZ as a function of time could be fitted reasonably well (a solid line in Figure 5) with an RMSD of 0.02 (Table S3).

The results of fitting in the presence of proline are presented in Figure 5B–E. Overall, the kinetic model simulated reasonably well the enhanced CBZ removal at different proline dosages. For initial proline dosages at 25.0 and 50.0 μM, the model accurately simulated the CBZ removal in 150 s, with modest deviation from the experimental data at the beginning stage (0–100 s), both with overall RMSD < 0.13 (Figure 5B,C). Furthermore, the model accurately simulated the CBZ removal kinetics with initial proline dosages of 100.0 and 200.0 μM, with RMSD values of 0.11 and 0.06, respectively (Figure 5D,E). In particular, the kinetic model reasonably agrees with the experimental results in that the fastest CBZ removal was achieved with 100.0 μM of proline, and the further increase of proline dosage to 200.0 μM led to a reduced enhancement.

Overall, the results supported that proline (and the resultant proline radical) activated Fe(VI) to produce highly reactive Fe(V), hence accelerating the overall CBZ removal. The kinetic modeling gave the rate constant for the reaction of Fe(V) with CBZ as (1.03 ± 0.21) × 10^6^ M^–1^ s^–1^, which is 6 orders of magnitude higher than that of Fe(VI) (Figure 5). This finding of the modeling is in agreement with the earlier estimated and experimentally determined rate constants for the reactions of Fe(VI) with pollutants using the premix pulse radiolysis technique.^71−73^ For example, the rate constants of Fe(V) with amino acids and carboxylic acids, determined using the premix pulse radiolysis technique, were determined to 3–5 orders of magnitude higher than that of Fe(VI).^60,61^ Interestingly, in the Fe(VI)–proline system, proline not only reduces Fe(VI) to produce Fe(V) (R4) but also competes with CBZ for Fe(V) (R7). In our study, the optimal proline dosage that led to the fastest CBZ degradation was 100.0 μM, while a further increase of the proline dosage to 200.0 μM could not further accelerate CBZ degradation, as demonstrated by kinetic modeling (Figure 5).

## Conclusions

The removal of CBZ by the Fe(VI)–amino acid system was higher than that by Fe(VI) alone in a mild alkaline medium. The most significant increase was found in the Fe(VI)–proline system. The kinetics of the reaction between Fe(VI) and amino acids suggested the role of the rate for the formation of a highly reactive intermediate, Fe(V), which was determined to be the highest for proline. The degradation of CBZ had no direct positive correlation with the rate constants of the reactions of Fe(VI) with amino acids, indicating that the possible influence of the cyclic structure of proline increased the removal of CBZ by the Fe(VI)–proline system. The kinetic modeling of degradation of CBZ at varied concentrations of proline could estimate the rate constants of Fe(V) with CBZ, which was found to be consistent with the known literature on the order of its reactivity with organic compounds being higher than that of Fe(VI). Overall, this study provides new insight that natural compounds such as amino acids may be combined with Fe(VI) to increase the removal efficiency for micropollutants of concern in water treatment.

## Supporting Information Available

HPLC condition to analyze trimethoprim, sulfamethoxazole, PMSO, and PMSO_2_ (Table S1); rate constants for the reactions of Fe(VI) with amino acids (Table S2); RMSD obtained in kinetic modeling (Table S3); and molecular structures of the studied amino acid (Figure S1) (PDF)
