# Supplementary material for: Oxidation of Pharmaceuticals by Ferrate(VI)–Amino Acid Systems: Enhancement by Proline
Source: J Phys Chem A. 2023 Mar 2;127(10):2314–21. doi: 10.1021/acs.jpca.3c00134 (PMC10848263; doi:10.1021/acs.jpca.3c00134)
Supplement: Supplementary file 1 — jp3c00134_si_001.pdf [file jp3c00134_si_001.pdf]

Supporting Information

Oxidation of Pharmaceuticals by Ferrate(VI)-Amino Acids System: Enhancement by Proline

Virender K. Sharma[1*], Junyue Wang[2], Mingbao Feng[1], and Ching-Hua Huang[2*]

[1]Department of Environmental and Occupational Health, School of Public Health,

Texas A&M University, College Station, Texas, 77843-8371, USA

vsharma@tamu.edu

[2]School of Civil and Environmental Engineering, [4]Georgia Institute of Technology, Atlanta, GA 30332, USA, ching-hua.huang@ce.gatech.edu

Table S1. HPLC conditions of SDM, TMP, and PMSO/$PMSO_2$.

Compound	Mobile phase Methanol/water	Flow rate (mL/min)	UV_{max} (nm)	Retention time (min)
SDM	50:50	0.8	268	6.837
TMP	35:65	1.0	271	4.197
PMSO	70:30	1.0	230	16.747
$PMSO_2$	70:30	1.0	215	18.324

Table S2. The second-order rate constant (k, M^{-1} s^{-1}) for the reactivity of Fe(VI) with amino acid in borate buffer at pH 9.0.

Amino Acid	Symbol	k, M^{-1} s^{-1}
Glycine	Gly	21.2±0.2
Alanine	Ala	10.2±0.1
Leucine	Leu	12.2±0.1
Serine	Ser	15.0±0.3
Asparagine	Asn	17.7±0.3
Glutamic Acid	Glu	7.6±0.1
Phenylalanine	Phe	13.7±0.2
Histidine	His	28.5±0.2
Proline	Pro	71.0±0.2

Table S3. RMSD values for kinetic modeling. ((Experimental conditions: $[CBZ]_0$ = 5.0 μM, $[Fe(VI)]_0$ = 100.0 μM, pH = 9.0 buffered by 10.0 mM Na_2HPO_4).

[Proline], μM	RMSD
**0**	0.01
**25**	0.13
**50**	0.12
**100**	0.11
**200**	0.06

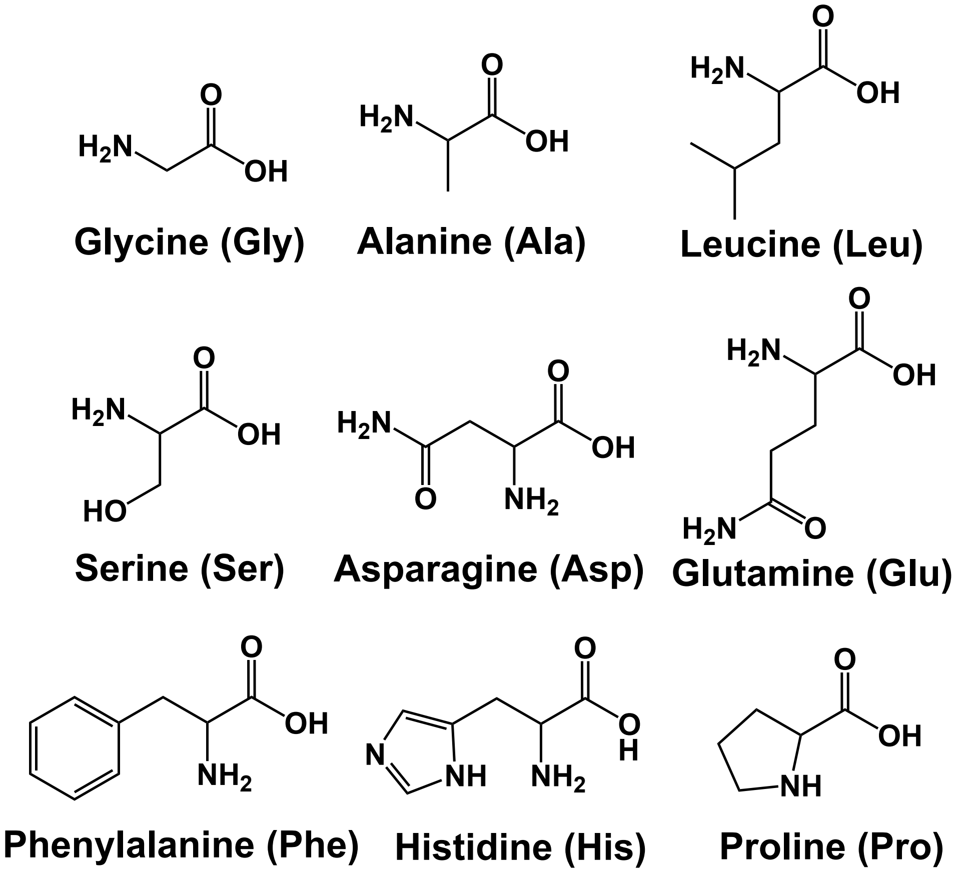

Figure S1. Studied amino acids in the Fe(VI)/amino acids (AA) system.
